# Muscle bursting and corticomotor excitability mark impaired impulse control in Parkinson’s disease

**DOI:** 10.1038/s41531-025-01207-5

**Published:** 2025-12-23

**Authors:** Aliya C. M. Warden, Craig J. McAllister, Damian Cruse, Ben Wright, Hayley J. MacDonald

**Affiliations:** 1 School of Sport, Exercise and Rehabilitation Sciences, College of Life and Environmental Sciences, University of Birmingham, Birmingham, UK; 2 Centre for Human Brain Health, University of Birmingham, Birmingham, UK; 3 School of Psychology, College of Life and Environmental Sciences, University of Birmingham, Birmingham, UK; 4 Department of Neurology, Queen Elizabeth Hospital Birmingham, Birmingham, UK; 5 Department of Biological and Medical Psychology, University of Bergen, Bergen, Norway

**Keywords:** Basal ganglia, Parkinson's disease, Neurophysiology, Human behaviour

## Abstract

Dopamine agonists in Parkinson’s disease increase the risk of impulse control disorders, yet the underlying neural mechanisms remain unclear. This study aimed to identify objective neurophysiological markers of impaired impulse control in Parkinson’s linking to impulsivity problems. Nineteen people with Parkinson’s (PwPD) on ropinirole and 18 healthy older adults performed an impulse control task requiring response withholding and inhibition. Transcranial magnetic stimulation was delivered, with corticomotor excitability (CME) and muscle bursts measured via electromyography. During response withholding, PwPD showed early relative increases in CME and more frequent dysfunctional muscle bursts, leading to more variable responses. During response inhibition, PwPD exhibited reduced CME suppression and increased muscle bursting, leading to delayed inhibition which was associated with problematic impulsive behaviours. The neurophysiological measures were associated with more advanced disease and may serve as early, objective markers of impulse control dysfunction, with future work required to assess their utility in predicting impulsive disorders.

## Introduction

Parkinson’s disease (PD) is a neurodegenerative disorder affecting 1–2 per 1000 people, with incidence rising sharply after 65^1,2^. While PD is characterised by motor symptoms such as bradykinesia, tremor, and rigidity, non-motor symptoms are also common, including impulse control disorders (ICDs). Dopamine agonists (DAs), especially ropinirole and pramipexole, are effective for motor symptoms but strongly associated with ICDs^3,4^ (~ 42% incidence)^5^. This susceptibility to ICDs has been linked to the dopamine overdose hypothesis^6,7^, in which medication induces a hyper-dopaminergic state in relatively intact neural circuitry, including overstimulation of D_3_ receptor-mediated reward pathways^8^. These ICD behaviours—pathological gambling, binge eating, compulsive shopping, and hyper-sexuality—can severely impact quality of life, relationships, and financial stability^9,10^. Currently, no objective method exists to identify those at risk.

Impulsivity encompasses two main domains: impulsive choice (impaired decision-making) and impulsive action (inability to suppress prepotent responses)^11^. Impulsive action is typically assessed through response inhibition paradigms such as the Go/No-Go and Stop-Signal tasks, which require withholding and cancellation of externally cued responses, respectively. PD-related impairments have been documented across both tasks^12–17^, including under DA treatment and before ICDs manifest^11,18^. Although there are some contrasting reports of intact response withholding and inhibition in PD^16,19^. People with PD (PwPD) have particular difficulty with internally generated, anticipatory responses, likely due to reduced preparatory suppression—corticospinal inhibition that typically prevents premature response initiation^20,21^.

Control of such anticipated responses can be examined using the Anticipatory Response Inhibition Task (ARIT) which is becoming increasingly popular^22^. In Go trials, response withholding must be proactively maintained and then strategically released to initiate movement synchronising with a stationary target. In contrast, Stop trials require reactive inhibition of the prepared response, triggered by the presentation of the stop-signal. Therefore, the ARIT provides insight into both proactive and reactive aspects of inhibitory control, which interact dynamically to enable adaptive behavioural control^23^. To our knowledge, only one study has applied the ARIT in PD^24^, demonstrating that poorer response inhibition performance was associated with a higher frequency of ICD behaviours on DA medication.

The neurophysiological mechanisms underlying impaired performance in the ARIT for PwPD remain unclear. Investigating these mechanisms could help to identify objective markers of impaired impulse control associated with everyday impulsive behaviour. For instance, electromyography recordings during the ARIT suggest that PwPD are more likely than age-matched controls to exhibit ineffective bursts of muscle activity when withholding the planned response in Go trials (see ref. ^25^). It is possible that this reflects dysfunction within the inhibitory control network, specifically preparatory suppression, and warrants further investigation in a full cohort.

Bursts of muscle activity during Stop trials also provide insight into underlying neurophysiological mechanisms; in this case, during the reactive stopping process. In successful Stop trials, no behavioural response occurs, as the prepotent movement is successfully inhibited. However, participants may still generate sub-threshold muscle activation, initiating but subsequently cancelling the motor response before sufficient force is generated to carry out the action. The latency of these bursts relative to the stop-signal (CancelTime) directly indexes action stopping at a single-trial level. These partial muscle bursts occur at rates of ~30–35% in healthy adults performing the ARIT^26,27^, and can be observed more frequently in clinical groups with basal ganglia dysfunction (e.g., focal dystonia)^28^. Overall, we propose muscle bursting during both response withholding and inhibition as a physiological marker of inhibitory control in PwPD.

To better understand muscle-bursting activity in PD, it is essential to explore further upstream in the motor control pathway. Many studies have applied single-pulse transcranial magnetic stimulation (TMS) over the motor cortex (M1) to record changes in corticomotor excitability (CME) during impulse control tasks in healthy adults (see ref. ^29^ for a review)^30,31^. Specifically in the ARIT, following a stable baseline period, CME increases around 250–175 ms before the target and remains elevated in Go trials^32–34^. In contrast, successful response inhibition is associated with a reduction in CME 140–175 ms following stop-signal presentation^33,35^. To our knowledge, no studies have investigated how CME fluctuations in PwPD compare to the typical pattern observed during response inhibition tasks.

Overall, the neural mechanisms underlying impaired impulse control in PwPD remain unclear, despite a high prevalence of destructive ICDs in this population. The present study aimed to identify objective markers of impaired impulse control in PD by examining changes in muscle bursting and CME measures during response withholding and inhibition. Dopamine overdose effects may disrupt the balance of excitation and inhibition within fronto-basal ganglia circuitry^36,37^, favouring facilitatory drive and weakening braking of downstream targets^38,39^. We therefore predicted that the widely hypothesised excessive dopaminergic tone linked to inhibitory problems on DAs would weaken inhibitory gating of CME, triggering increased muscle bursting and impairing response control. Our specific hypotheses were as follows; compared to healthy older adults, PwPD on the DA ropinirole would show: (1) an impaired ability to (a) withhold and (b) inhibit the prepared lift response in the ARIT; (2) more ineffective muscle bursting activity during (a) response withholding and (b) response inhibition; (3) (a) increased CME during response withholding and (b) reduced CME suppression during response inhibition. A further aim was to investigate whether these objective markers were associated with everyday impulsive behaviour. The use of such objective measures could facilitate early identification of individuals at heightened risk for ICD development, perhaps even prior to the initiation of dopaminergic therapy, enabling more informed treatment planning. Our final hypothesis was therefore that (4) PwPD on ropinirole would show a link between muscle bursting and CME changes with task performance and self-reported impulsivity.

## Results

### Demographic and clinical data

Following data collection, two PwPD were excluded due to outlying behavioural values (lift-time/stop-signal reaction time [SSRT] identified via the box-plot method). One PwPD and one HC were excluded due to low signal-to-noise ratio in the electromyography (EMG) recording. One HC was excluded due to unreliable baseline motor evoked potentials (MEPs).

Table 1 provides a summary of demographic and clinical data, with group comparisons where applicable. PwPD were younger than HCs; therefore, age was included as a covariate in analyses. Symptom severity ranged from mild to moderate (MDS-UPDRS III: 9–55)^40^ and disease duration from 1 to 9.8 years. On average, participants took ropinirole 4.3 ± 3.7 h before the study session began. Two participants were on ropinirole as primary therapy, 16 were also on levodopa, seven on rasagiline, two on safinamide, two on entacapone, one on opicapone and one on rotigotine.

**Table 1:** Participant demographic, cognitive and clinical variables separated by study group

	PwPD (*n* = 19)	HCs (*n* = 18)	*p*-value
Age (years)	64.1 ± 7.0	69.1 ± 5.6	*p* = 0.022*
Sex (F:M)	8:11	9:9	*p* = 0.677†
Handedness (L:R)	3:16	2:16	*p* = 0.630†
MoCA (/30)	27.1 ± 1.7	27.6 ± 1.5	*p* = 0.351
BIS-11 (%)	52.4 ± 7.2	49.0 ± 8.0	*p* = 0.085
QUIP-RS (%)	26.1 ± 14.3	17.4 ± 11.6	*p* = 0.025*
Symptom laterality (L:R)	8:11	-	-
Disease duration (years)	5.8 ± 2.3	-	-
MDS-UPDRS (total)	50.7 ± 21.0	-	-
MDS-UPDRS I	11.3 ± 5.9	-	-
MDS-UPDRS II	12.1 ± 7.1	-	-
MDS-UPDRS III	27.4 ± 11.7	-	-
H&Y stage	1.7 ± 0.6	-	-
Ropinirole dose (mg)	10.7 ± 5.3	-	-
LEDD (mg/day)	671.2 ± 319.3	-	-
Time on medication (years)	3.8 ± 2.1	-	-

Values represent means ± SD, unless otherwise indicated. Handedness and symptom laterality were self-reported. BIS-11: Barratt Impulsiveness Scale, HCs: healthy controls, H&Y: Hoehn and Yahr, QUIP-RS: Questionnaire for Impulsive-Compulsive Disorders in Parkinson’s Disease-Rating Scale, LEDD: levodopa equivalent daily dose^90^, MoCA: Montreal Cognitive Assessment, PwPD: people with Parkinson’s disease, MDS-UPDRS: Movement Disorder Society-sponsored revision of the Unified Parkinson’s Disease Rating Scale (tested while ‘on’ medication).

*p < 0.05, †chi-square test. All other *p*-values reported from independent-samples *t*-test.

### Behavioural data

**Fig. 1:**
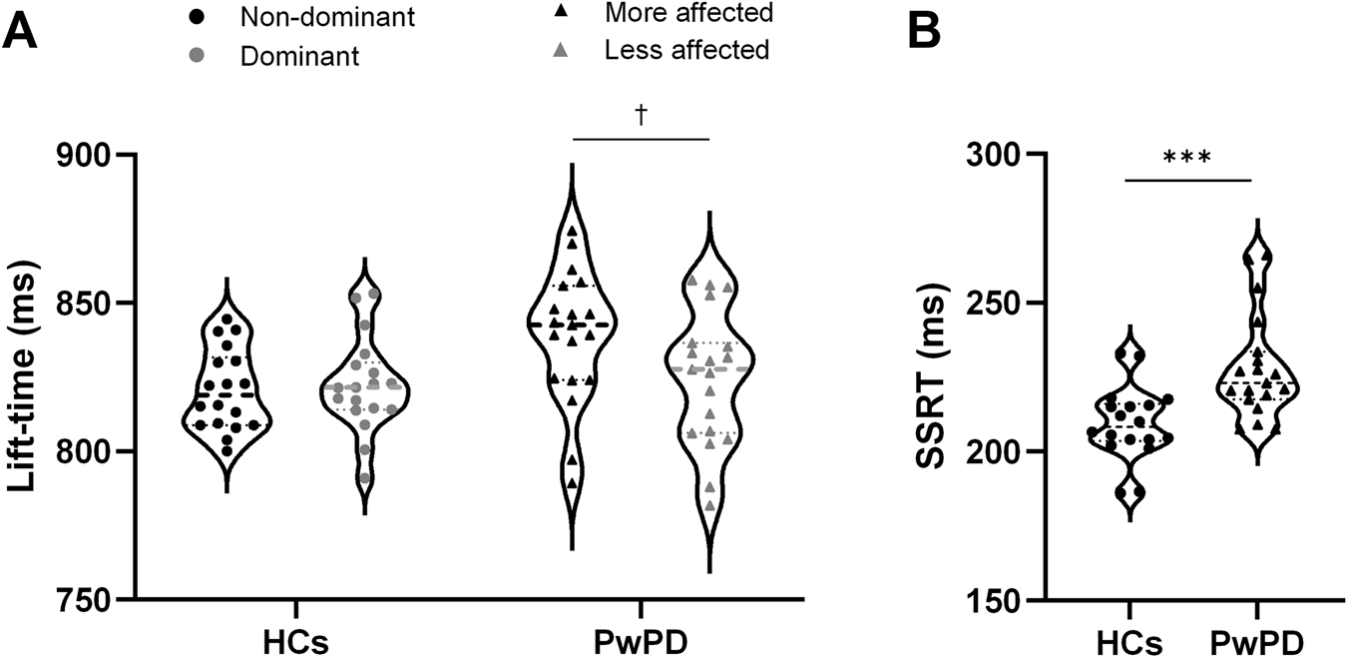
Behavioural results across Go and Stop trials. Across Go trials, there was no main effect of Group on mean lift-times between people with Parkinson’s disease (PwPD; 830.9 ± 24.2 ms) and healthy controls (HCs; 821.5 ± 14.6 ms) (**A**), although lift time variability was greater in PwPD (*p* = 0.008) and linear mixed-effects modelling revealed later lift-times in the more affected hand in PwPD (837.2 ± 24.7 vs. 824.5 ± 22.5 ms, † *p* < 0.001). Across successful Stop trials, stop-signal reaction time (SSRT) was longer in PwPD (black triangles; 228.1 ± 17.6 ms) versus HCs (black circles; 209.3 ± 12.4 ms, *** *p* < 0.001) (**B**). The median and quartiles are indicated with dashed and dotted lines, respectively.

The mean lift-times of both PwPD (830.9 ± 24.2 ms) and HCs (821.5 ± 14.2 ms) were slightly after the target on Go trials (Fig. 1A). There was no main effect of Hand (*F*_1,36_ = 1.80, *p* = 0.188), or Group (*F*_1,35_ = 3.40, *p* = 0.074) or Hand × Group interaction (*F*_1,36_ = 3.06, *p* = 0.089). However, there was a main effect of Group (*F*_1,35_ = 7.92, *p* = 0.008, η_p_^2^ = 0.19, 90% CI [0.03–0.36]) on lift-time coefficient of variation, with PwPD exhibiting more variable lift-times than HCs (5.8 ± 1.3 vs. 4.8 ± 0.8, respectively), indicating poorer control over response withholding. This effect remained when age was included as a covariate (*F*_1,34_ = 11.11, *p* = 0.002, η_p_^2^ = 0.25, 90% CI [0.06–0.42]). There was no main effect of Hand (*p* = 0.398) or Hand × Group interaction (*p* = 0.609) on lift-time variability. Linear mixed-effects model (LMM) analyses confirmed the between-group differences in lift-time variability and further identified delayed responses in the more affected hand in PwPD (see Supplementary Material and Fig. 1A).

On Stop trials, stopping success rates between 49–53% (51.2 ± 0.9%) indicate that the stop-signal delay (SSD) staircasing procedure was effective. Staircased SSD values were significantly earlier in PwPD (598.7 ± 17.6 ms) than HCs (610.0 ± 15.2 ms, *p* = 0.025), indicating more time was needed to inhibit the response. Further, as hypothesised, PwPD demonstrated significantly longer SSRTs (228.1 ± 17.6 ms) versus HCs (209.3 ± 12.4 ms, *p* < 0.001, Fig. 1B), reflecting worse response inhibition. This effect was maintained after controlling for age (*p* < 0.001).

### Muscle bursting

**Fig. 2:**
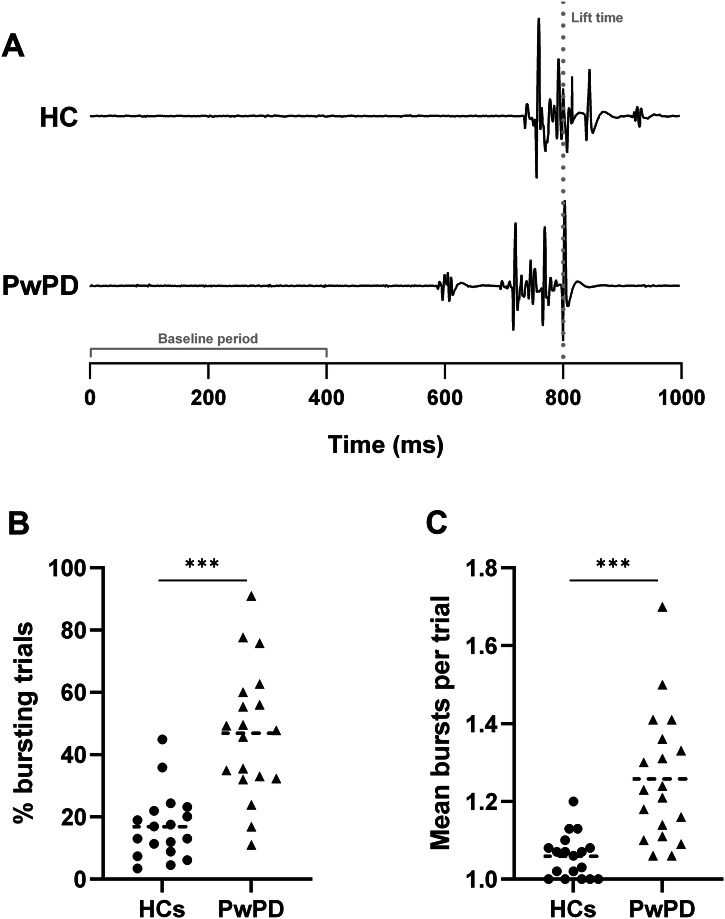
Premature bursting results on Go trials. **A** shows representative electromyographic traces for each group, which demonstrate a premature muscle burst (~ 600 ms) prior to the main lift response in a person with Parkinson’s disease (PwPD; grey dotted line indicates lift-time). **B** shows a higher percentage of Go trials with ≥1 premature burst(s) across both sides in PwPD (black triangles; 46.9 ± 21.0%) compared to healthy controls (HCs; black circles; 16.9 ± 10.8%; *** *p* < 0.001). **C** shows a greater frequency of bursts within these trials in PwPD (black triangles; 1.26 ± 0.17 vs. 1.06 ± 0.06; *** *p* < 0.001).

**Fig. 3:**
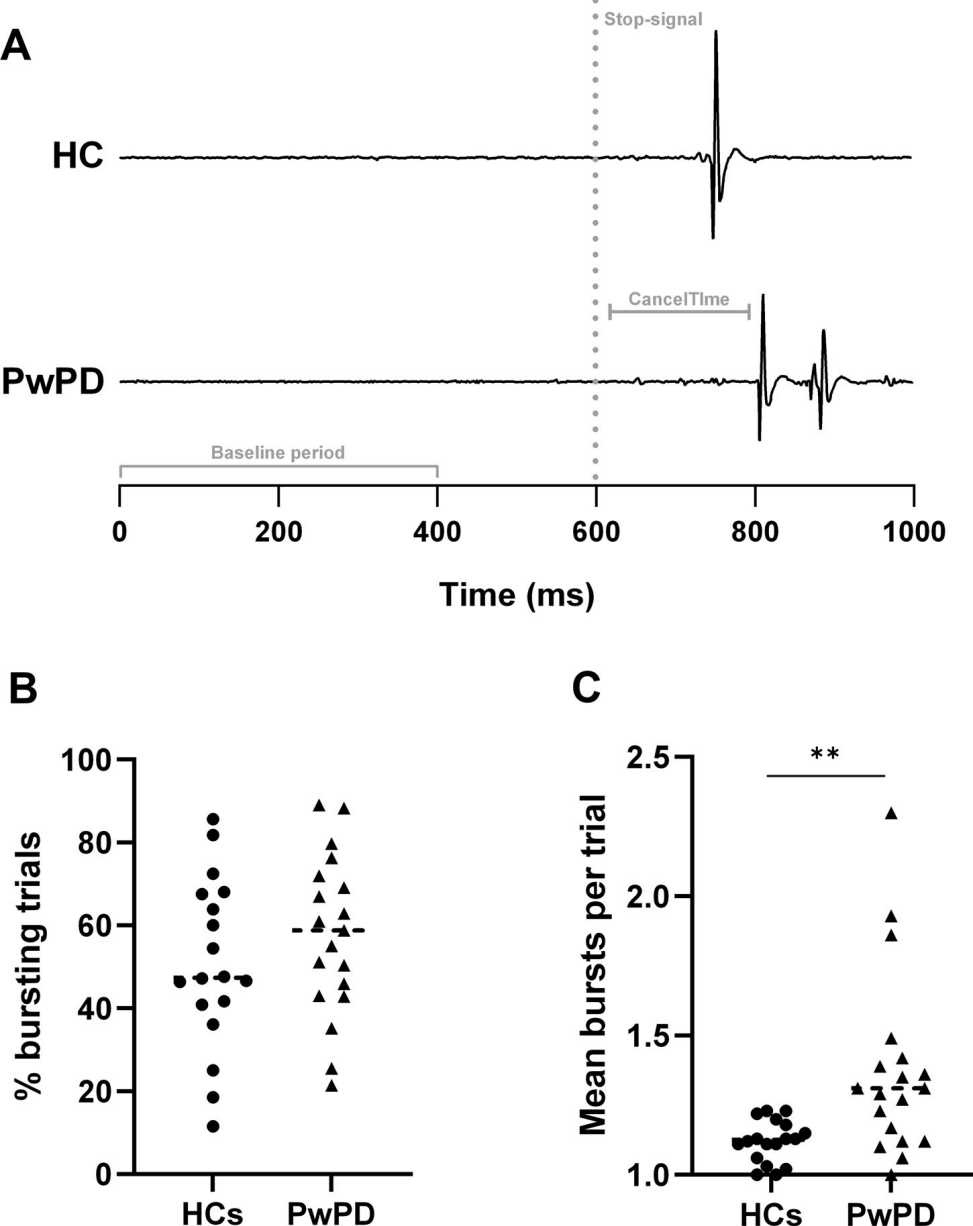
Partial bursting results on successful Stop trials. **A** shows representative electromyographic traces for each group, which demonstrate partial muscle bursts in both a healthy control (HC) participant and a person with Parkinson’s disease (PwPD) following the stop-signal (grey dotted line) around when the response would have occurred (~ 750–800 ms). **B** shows both groups exhibited ≥1 partial muscle burst(s) in >50% of trials. **C** shows a greater frequency of bursts within these trials in PwPD (black triangles; 1.37 ± 0.33 vs. 1.12 ± 0.08; ***p = 0.001) across both sides.

Both groups demonstrated premature muscle bursts prior to the lift response on Go trials (Fig. 2A) and partial bursts when the lift would have occurred on successful Stop trials (Fig. 3A). Mean premature burst onset ranged from 90–178 ms before the target in HCs and 85–258 ms before the target in PwPD. Pillai’s Trace revealed a significant multivariate effect of Group on the combined bursting characteristics (incidence, frequency, rate, amplitude, duration) for both premature (*V* = 0.53, *F*_5,31_ = 6.88, *p* < 0.001) and partial bursts (*V* = 0.35, *F*_5,31_ = 3.38, *p* = 0.015). Follow-up univariate analyses of variance (ANOVAs) are described below.

As hypothesised, PwPD demonstrated greater premature muscle burst activity than HCs on Go trials. There was a significant effect of Group on premature burst rate (*F*_1,35_ = 31.70, *p* < 0.001, η_p_^2^ = 0.48, 90% CI [0.26–0.61]), with a higher rate in PwPD (0.62 ± 0.36) compared to HCs (0.18 ± 0.13). This measure reflected a higher incidence of Go trials with ≥1 premature burst(s) in PwPD (46.9 ± 21.0%) compared to HCs (16.9 ± 10.8%; *F*_1,35_ = 30.61, *p* < 0.001, η_p_^2^ = 0.47, 90% CI [0.25–0.60]; Fig. 2B) and a greater frequency of bursts within these trials (PwPD: 1.26 ± 0.17; HC: 1.06 ± 0.06; *F*_1,35_ = 26.06, *p* < 0.001, η_p_^2^ = 0.43, 90% CI [0.21–0.57]; Fig. 2C). There were no group differences in burst amplitude (*p* = 0.361) or duration (*p* = 0.953). In PwPD, premature burst incidence (*p* = 0.094) and frequency (*p* = 0.091) were comparable between the more and less affected hands.

Both groups had a similar proportion of successful Stop trials (PwPD: 57.6 ± 19.3%; HC: 50.9 ± 20.6%; *p* = 0.279) containing ≥1 partial muscle burst(s) (Fig. 3B). However, these bursts occurred more frequently within-trials in PwPD (1.37 ± 0.33) compared to HCs (1.12 ± 0.08; *F*_1,35_ = 11.92, *p* = 0.001, η_p_^2^ = 0.25, 90% CI [0.07–0.42]; Fig. 3C). The main effect of Group on burst rate did not reach statistical significance (PwPD: 0.81 ± 0.37; HC: 0.58 ± 0.26; *F*_1,35_ = 3.83, *p* = 0.058). Again, there were no group differences in amplitude (*p* = 0.401) or duration (*p* = 0.066), and partial burst frequency was comparable between the two sides in PwPD (*p* = 0.359).

In accordance with our SSRT findings, CancelTime was longer in PwPD (184.5 ± 44.6 ms after the stop-signal) than HCs (160.1 ± 14.0 ms; *p* = 0.015), reflecting delayed inhibition latency. As expected, there was a positive correlation between CancelTime and SSRT (*r* = 0.617, *p* < 0.001).

### Corticomotor excitability

**Fig. 4:**
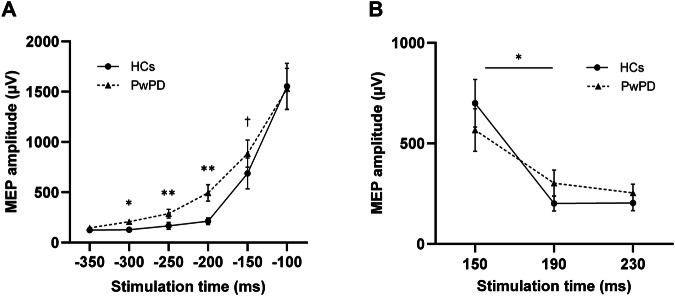
Corticomotor excitability results across Go and successful Stop trials. In Go trials (**A**), while corticomotor excitability (CME), measured via amplitude of motor evoked potentials (MEPs) increased across both groups in anticipation of the target, people with Parkinson’s disease (PwPD) demonstrated an earlier increase in CME from −300 ms to −150 ms (black triangles). Between-group differences at each timepoint are indicated with *p < 0.05; **p < 0.01; †*p* < 0.05 with age as a covariate. In successful Stop trials (**B**), while both groups demonstrated a reduction in CME, PwPD exhibited a smaller drop in CME from 150 ms to 190 ms (black triangles, *p = 0.025). Values are mean ± standard error. MEPs were measured from the non-dominant (HC) or more affected (PwPD) first-dorsal interosseous (FDI) muscle.

Stimulation intensity was set at 48.7 ± 8.3% to obtain baseline MEPs in the 200–400 µV range. For Go trials (Fig. 4A), CME increased in both groups as the target approached (*F*_5,175_ = 182.36, *p* < 0.001, η_p_^2^ = 0.84, 90% CI [0.80–0.86]). However, a Group × Stimulation Time interaction (*F*_5,175_ = 3.84, *p* = 0.003, η_p_^2^ = 0.10, 90% CI [0.02–0.15]) revealed that CME increased earlier in PwPD at −300 ms (207.7 ± 107.5 μV vs. 128.3 ± 82.6 μV, *p* = 0.021) and remained above HCs until −200 ms (494.4 ± 359.2 μV vs. 213.0 ± 140.8 μV, *p* = 0.003). The observed effects became more pronounced after controlling for age (*F*_5,170_ = 5.05, *p* < 0.001, η_p_^2^ = 0.13, 90% CI [0.04–0.19]). PwPD additionally demonstrated higher CME than HCs at −150 ms when age was added as a covariate (885.0 ± 594.1 μV vs. 688.2 ± 654.5 μV, *p* = 0.027). Both groups had comparable CME values at the final timepoint (−100 ms). These results were further confirmed with LMM analyses (see Supplementary Material).

For pre-trigger root-mean-squared (rms)EMG on Go trials, there was no main effect of Group (*p* = 0.908) or Group × Stimulation Time interaction (*p* = 0.139). There was a main effect of Stimulation Time (*F*_5,175_ = 59.07, *p* < 0.001, η_p_^2^ = 0.63, 90% CI [0.55–0.67]), with rmsEMG rising −200 ms relative to the target in both groups.

For successful Stop trials, there was a main effect of Stimulation Time (*F*_2,70_ = 44.92, *p* < 0.001, η_p_^2^ = 0.56, 90% CI [0.42 – 0.64]), with an overall reduction in MEP amplitude between 150 ms (631.3 ± 479.9 μV) and both 190 ms (252.7 ± 233.6 μV, *p* < 0.001) and 230 ms (229.3 ± 177.1 μV, *p* < 0.001) following the stop-signal. There was no main effect of Group (*p* = 0.961), however, there was a Group × Stimulation Time interaction (*F*_2,70_ = 4.28, *p* = 0.025, η_p_^2^ = 0.11, 90% CI [0.01 – 0.22]), with PwPD demonstrating a smaller drop in MEP amplitude between 150–190 ms (264.8 ± 338.6 μV) following the stop-signal compared to HCs (498.7 ± 413.0 μV, *p* = 0.034; Fig. 4B). The above effects mostly remained consistent when age was included as a covariate, although the Group × Stimulation Time interaction trended towards significance (*F*_2,68_ = 3.37, *p* = 0.051, η_p_^2^ = 0.09, 90% CI [0.002 – 0.19]). These results were further confirmed with LMM analyses (see Supplementary Material).

For pre-trigger rmsEMG on successful Stop trials, there was a main effect of Stimulation Time (*F*_2,70_ = 73.91, *p* < 0.001, η_p_^2^ = 0.68, 90% CI [0.56 – 0.74]), with an overall reduction at 230 ms (17.2 ± 18.1 μV) compared to 150 ms (52.5 ± 59.8 μV, *p* < 0.001) and 190 ms (57.4 ± 65.1 μV, *p* < 0.001). There was also a Group × Stimulation Time interaction (*F*_2,70_ = 4.04, *p* = 0.022), with higher rmsEMG values in HCs than PwPD at 190 ms (57.4 ± 51.3 μV vs. 47.9 ± 67.9 μV, *p* = 0.02) and 230 ms (74.3 ± 66.9 μV vs. 41.4 ± 60.8 μV, *p* = 0.009). Crucially, this interaction revealed an inverse relationship between rmsEMG and CME changes, with HCs exhibiting *higher* rmsEMG alongside a larger *decrease* in MEP amplitude compared to PwPD. Therefore, there was no group difference in pre-trigger muscle activity across 150–190 ms that could account for the difference in MEP amplitudes.

### Regression modelling

In the regression models described below, the assumption of no multicollinearity was met, as indicated by a Variance Inflation Factor (VIF) of 1 for all predictors.

To model response withholding performance, lift-time variability in Go trials was included as the outcome variable. Predictor variables were premature burst rate, mean CME across the timepoints showing increased excitability in PwPD (−300 ms, −250 ms, and −200 ms), and their interaction. Premature burst rate interacted with CME to predict lift-time variability (F_1,17_ = 5.59, *p* = 0.03), accounting for ~20% of the variance (R^2^ 0.248, adjusted R^2^ 0.203). A simple slopes analysis indicated that increased premature burst rates combined with high CME levels were associated with greater lift-time variability (*p* = 0.038, *r* = 0.836).

To model response inhibition performance, SSRT and CancelTime were included as outcome variables. Predictor variables were partial burst frequency, CME difference score (150–190 ms), and their interaction. In the SSRT model, none of the predictor variables met the statistical entry criteria (*p* < 0.05) for inclusion. However, partial burst frequency emerged as a significant predictor of CancelTime (F_1,17_ = 38.65, *p* < 0.001. *β* = 0.833), accounting for ~68% of the variance (R^2^ 0.695, adjusted R^2^ 0.677). Specifically, a 10% increase in partial burst frequency was associated with a ~ 8.3% increase in CancelTime.

Four stepwise linear regressions were conducted to model self-reported impulsivity via the Barratt Impulsiveness Scale (BIS-11) and Questionnaire for Impulsive-Compulsive Disorders in Parkinson’s Disease Rating Scale (QUIP-RS) in HCs and PwPD separately. As per the previous models for task performance, neurophysiological predictor variables included premature burst rate and mean CME in Go trials, and partial burst frequency, CME difference score and CancelTime in Stop trials, as well as multiple interaction terms capturing within- and between-trial dynamics:

$$\begin{array}{l}y\left(Self-report\,Impulsivity\right)\\ \,\,\,\,\,\,\,={\beta }_{0}\left(intercept\right)+{\beta }_{1}Premature\,Burst\,Rate+{\beta }_{2}Go\,CME\\ \,\,\,\,\,\,\,\,\,\,\,\,\,\,\,\,\,+\,{\beta }_{3}Partial\,Burst\,Frequency+{\beta }_{4}Stop\,CME+{\beta }_{5}Cancel\,Time\\ \,\,\,\,\,\,\,\,\,\,\,\,\,\,\,\,\,+\,{\beta }_{6}Premature\,Burst\,Rate* Go\,CME+{\beta }_{7}Partial\,Burst\,Frequency\\ \,\,\,\,\,\,\,\,\,\,\,\,\,\,\,\,\,*\, Stop\,CME+{\beta }_{8}Partial\,Burst\,Frequency* Cancel\,Time+{\beta }_{9}Stop\,CME\\ \,\,\,\,\,\,\,\,\,\,\,\,\,\,\,\,\,*\, Cancel\,Time+{\beta }_{10}Premature\,Burst\,Rate* Partial\,Burst\,Frequency\\ \,\,\,\,\,\,\,\,\,\,\,\,\,\,\,\,+\,{\beta }_{11}Go\,CME* Stop\,CME+{\beta }_{12}Partial\,Burst\,Frequency* Stop\,CME\\ \,\,\,\,\,\,\,\,\,\,\,\,\,\,\,\,\,*\, Cancel\,Time+\varepsilon \end{array}$$

In HCs, premature burst rate interacted with Go CME to predict QUIP-RS score (F_1,17_ = 9.23, *p* = 0.008), accounting for ~33% of the variance (R^2^ 0.366, adjusted R^2^ 0.326). A simple slopes analysis indicated that increased premature burst rates at higher CME levels were associated with higher QUIP-RS scores (*p* = 0.05, *r* = 0.666), indicating increased impulsive behaviours. In addition, partial burst frequency interacted with Stop CME to predict BIS-11 score (F_1,17_ = 6.87, *p* = 0.019), accounting for ~26% of the variance (R^2^ 0.300, adjusted R^2^ 0.257). A simple slopes analysis indicated increased partial bursting in conjunction with reduced CME suppression was associated with higher BIS-11 scores, indicating increased trait impulsivity, although this did not reach statistical significance (*p* = 0.071, *r* = 0.627).

While links were established between neurophysiological measures and self-reported impulsivity in HCs, none of the predictor variables met the statistical entry criteria (*p* < 0.05) for inclusion in the final BIS-11 or QUIP-RS models for PwPD. To check the validity of the reported QUIP-RS scores, we examined whether a previously reported association between response inhibition performance and clinical impulsivity could be replicated^24^ by incorporating SSRT into the existing model. Indeed, the resulting model indicated that SSRT was a significant predictor of QUIP-RS score (*F*_1,17_ = 9.22, *p* = 0.007, *β* = 4.859), accounting for ~31% of the variance (*R*^2^ 0.352, adjusted *R*^2^ 0.313). Specifically, a 10% increase in SSRT was associated with a ~ 48.6% increase in QUIP-RS score.

Follow-up stepwise regression analyses tested for potential relationships between clinical variables (disease duration, MDS-UPDRS total score, Hoehn and Yahr stage, ropinirole dose, LEDD and time on medication) and behavioural/neurophysiological measures that showed group differences (premature burst rate, Go CME, lift-time variability, partial burst frequency, Stop CME, CancelTime and SSRT). These analyses revealed a significant interaction between symptom severity (MDS-UPDRS total) and overall medication dose (LEDD) in predicting several measures related to response inhibition, including partial burst frequency (*F*_1,17_ = 7.25, *p* = 0.015, adjusted *R*^2^ 0.258), CancelTime (*F*_1,17_ = 4.70, *p* = 0.045, adjusted *R*^2^ 0.171) and SSRT (*F*_1,17_ = 4.89, *p* = 0.041, adjusted *R*^2^ 0.178). A simple slopes analysis indicated that for those with greater symptom severity, higher medication doses were associated with increased partial bursting (*p* = 0.016, *r* = 0.895) and a trend toward longer CancelTime values (*p* = 0.086, *r* = 0.750). As expected, symptom severity and medication dose were positively correlated (*r* = 0.502, *p* = 0.014).

## Discussion

The present study generated several novel findings regarding the mechanisms underlying impaired impulse control in PwPD. We identified objective neurophysiological measures sensitive to deficits in response withholding and response inhibition in PD, which were associated with a more severe clinical profile. Compared to healthy older adults, PwPD exhibited both a higher rate of dysfunctional muscle bursts and an early transient increase in CME during Go trials, manifesting as more variable lift responses. PwPD also demonstrated a smaller reduction in CME and a higher frequency of muscle bursts on Stop trials which led to impaired response inhibition performance, that was itself predictive of more clinically problematic impulsive behaviours. We provide evidence that proactive and reactive inhibitory processes are compromised in PD at both a behavioural and neurophysiological level. Importantly, difficulties with general motor control in PD and background muscle activity were unable to account for our novel neurophysiological results. We discuss the theoretical and clinical implications of our findings below.

We confirmed an increased variability in ARIT lift responses for PwPD^41^, which has previously been shown on externally cued tasks^42–45^. Our results showed that a higher rate of premature muscle bursts (both within and across trials) at high CME levels was associated with the increased variability in lift responses in PwPD. Interestingly, however, the amplitude and duration of these premature bursts did not differ between groups. We therefore propose their occurrence alone serves as a marker of the state of the inhibitory control system, signalling less reliable response withholding resulting from impaired proactive inhibitory control in PwPD^46^. The functional significance of increased burst rate despite an unchanged bursting profile remains to be determined.

**Fig. 5:**
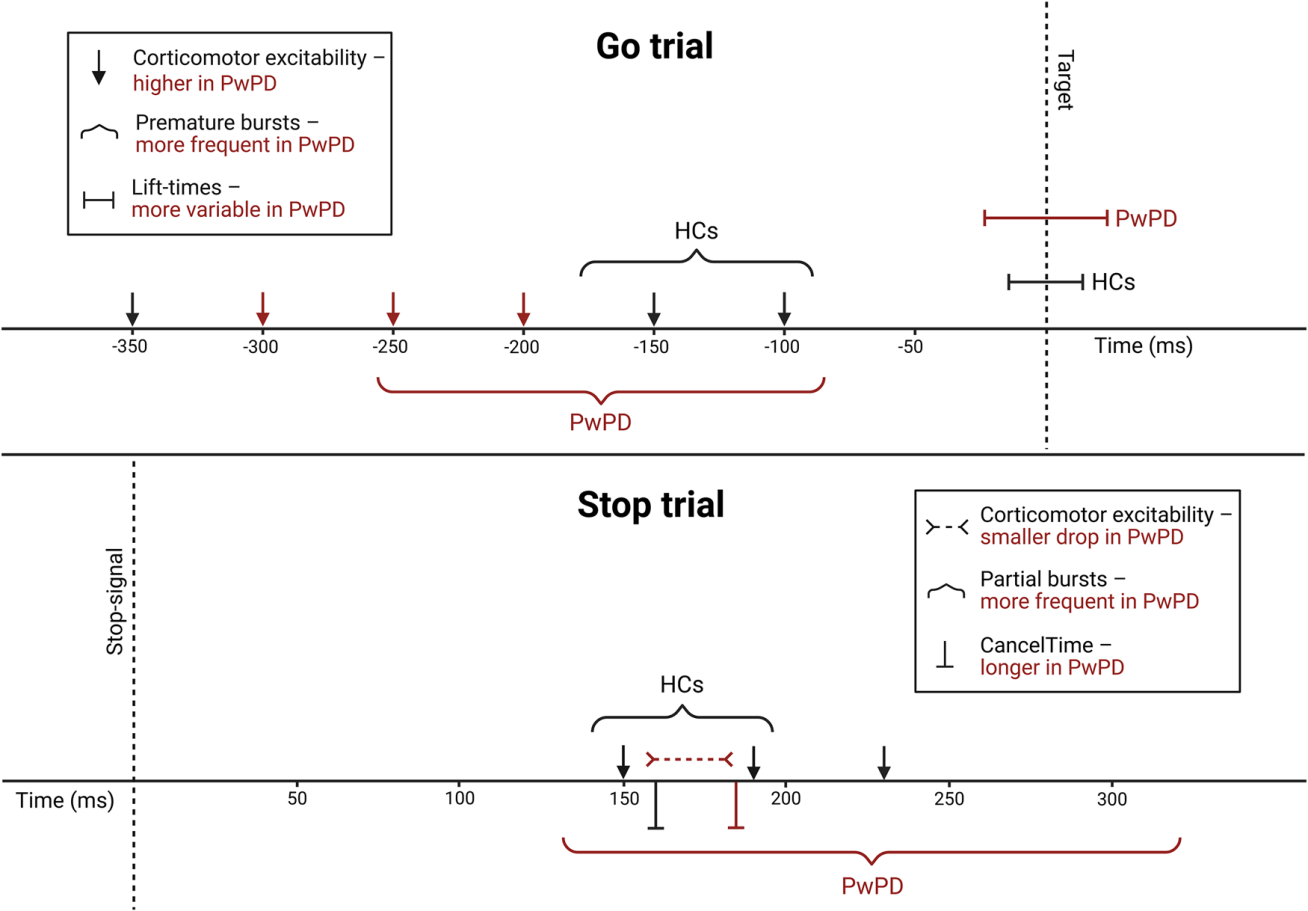
Results summary. A schematic of the relative timings of the corticomotor excitability, muscle bursting and behavioural results across Go and successful Stop trials. Group differences between people with Parkinson’s disease (PwPD) and healthy controls (HCs) are indicated in red.

While healthy older adults maintained a stable inhibitory state during response withholding (as per^32–34^) until ~150 ms before the target—at which point MEP amplitudes increased above baseline—PwPD demonstrated an early and transient rise in excitability above HCs. This shift in CME preceded the onset of premature muscle bursts (Fig. 5), perhaps reflecting impairment in preparatory suppression^20,21,47^, whereby elevated CME propagates through the motor system, triggering muscle bursting and thereby increasing response variability. This novel finding offers a more nuanced understanding of inhibitory control function, whereby insufficient gating in the motor system appears to directly impact response control. Such impairment could be mediated via dysfunctional activity in GABAergic inhibitory networks within M1^48–50^, a potential area for future research.

PwPD also demonstrated impaired inhibition of the anticipated response at both behavioural and neurophysiological levels. In accordance with previous research^13,14,17,51^, PwPD exhibited longer SSRTs compared to HCs, however, we are the first to show that the related measure of CancelTime was also longer in PwPD. A greater number of partial bursts within individual trials was associated with longer CancelTime values, indicative of fragmented motor suppression. Partial burst frequency is therefore a potential trial-by-trial indicator of reactive response inhibition, reflecting how ineffective engagement of the inhibitory control network in PD allows multiple bursts of muscle activity to emerge, thus delaying complete inhibition.

While a greater number of partial bursts occurred during response inhibition trials, the overall number of successful Stop trials exhibiting partial bursts was comparable between PwPD and HCs. This is not entirely surprising, as partial bursts have been observed in healthy adults across the Stop-Signal Task^26,52^ and ARIT^26,27^, although to a lesser extent. The increased prevalence (~ 50%) in our study may reflect age-related slowing of inhibition (see^53^ for a meta-analysis), potentially leading to greater difficulty in suppressing motor activity. Therefore, partial burst prevalence may not serve as a sensitive marker of PD-specific inhibitory deficits but rather reflect broader age-related declines in motor control.

While healthy older adults dynamically suppressed CME during successful stopping, consistent with findings in younger cohorts^33,35^, PwPD demonstrated a blunted inhibitory response that failed to sufficiently clamp down on CME in a timely manner. As pre-trigger muscle activity did not mirror the observed MEP changes, the attenuated CME suppression in PD reflects processes upstream of the alpha-motoneuron pool, likely within dopaminergic fronto-basal ganglia networks which show well-documented impairments in PD^54–56^. For instance, successful response inhibition is thought to originate via activation of the right inferior frontal gyrus and pre-supplementary motor area^57–59^ which are both prefrontal regions impacted by PD^60,61^. It is plausible that the results in the current study are mediated by deficits in top-down inhibitory signalling^62^ from disruption in these frontal regions, leading to disinhibition of the motor system in PwPD, demonstrated by reduced CME suppression, increased partial bursting and impaired stopping performance (Fig. 5). Future neuroimaging research could more clearly elucidate how motor-level and prefrontal cognitive-level contributions interact to shape inhibitory control in PwPD.

Of note, while neither muscle bursting or CME were linked with SSRT, more frequent partial bursts were associated with longer CancelTime values, suggesting that ineffective motor suppression contributes to delayed inhibition. It appears, therefore, that CancelTime and SSRT reflect distinct aspects of inhibitory control, despite being strongly correlated. SSRT provides an estimate of stopping performance based on average behavioural data (e.g., lift-times, SSD)^63^. Despite theoretical limitations^64–66^, longer SSRTs have been consistently associated with increased impulsivity across both clinical^14,24,67,68^ and non-clinical populations^69^. This association was further supported in the current study. Therefore, SSRT may serve as a broader measure of inhibitory control, relating more directly to real-world impulsive behaviours.

In contrast, CancelTime directly indexes stopping latency at the point where covert muscle activation begins to decrease on an individual-trial basis, isolating the neuromuscular mechanisms underlying response inhibition from potential experimental variations (e.g., pressing vs. releasing responses, button stiffness)^52^. Therefore, CancelTime may primarily reflect an active inhibition mechanism that blocks ongoing motor activity (e.g.,^70^,) which may not (yet) translate to broader impulsivity traits. However, it is important to note that CancelTime was measured using the peak of the earliest partial burst identified within each trial. PwPD exhibited a greater frequency of partial bursts within-trials, indicative of incomplete inhibition. As a result, CancelTime values reported in this study may underestimate the duration of the inhibitory process in PwPD, necessitating further investigation. Together, our findings underscore the importance of integrating both behavioural and neurophysiological measures when assessing impulse control, as they can capture distinct yet complementary aspects of the inhibition process.

While links were identified between our neurophysiological markers (elevated CME, increased muscle bursting) and self-report impulsivity (via QUIP-RS/BIS-11) in HCs across both response withholding and inhibition, no such links were established in PwPD. These discrepancies may reflect heterogeneity within the PD group, with wide variation in symptom presentation, disease severity and medication profile (e.g., types, doses). The specific effects of dopamine medication on impulse control behaviour in PwPD are mixed and likely occur on an individualised level according to patient characteristics (e.g., disease status, baseline behaviour), medication profiles and the employed task^11^. Indeed, our post-hoc regressions revealed that symptom severity interacted with overall medication dose to predict neurophysiological measures of response inhibition (partial bursting and CancelTime). This extends findings from a meta-analysis^14^, whereby longer disease duration predicted greater impulsive action deficits in PwPD on medication. Interestingly, while higher ropinirole doses have specifically been linked to impulsive behaviour^71^, no such association was found in the current study, suggesting that overall dopamine levels had a greater impact on impulse control performance. Taken together, our neurophysiological measures appear sensitive to early inhibitory dysfunction (as revealed in HCs), as well as progressive disease-related changes (as observed in PwPD).

The link between a more severe clinical profile and neurophysiological markers of impaired inhibition suggests that some individuals were shifted beyond the optimal dopamine range, particularly in circuitry that remains relatively intact^72^. Those with more progressed pathology may require higher doses to manage motor symptoms, at the cost of shifting mesocorticolimbic networks implicated in cognitive/limbic control into a hyperdopaminergic state, resulting in neurophysiological/behavioural impairment^6,73^. Clinically, these findings underscore the importance of individualised treatment strategies that consider the potential non-motor side effects of dopaminergic therapy. Although exploratory, these findings highlight neurophysiological markers such as partial bursting and CancelTime as potential early indicators of dopamine overdose effects on impulse control before the emergence of overt ICD behaviours, warranting further investigation.

Importantly, despite a range of symptom and medication profiles, we observed robust group effects across the applied behavioural and neurophysiological measures, reinforcing the presence of inhibitory control deficits across a heterogeneous PD sample. However, without comparison to PwPD who are medication-naïve (de novo) or temporarily withdrawn from dopaminergic treatment, we cannot disentangle medication effects from pathology-related inhibitory dysfunction. Further exploration of these measures in de novo PD would help determine whether the neurophysiological changes observed in the current study emerge prior to behavioural impairments early in the disease course^74^, with longitudinal follow-up as the disease progresses crucial to assess their predictive validity for future impulse control problems.

While our sample included a range of symptom severities (MDS-UPDRS III: 9–55), participants were limited to Hoehn and Yahr stages ≤2.5 and disease durations <10 years, limiting generalisability of the findings to more advanced PD. Notably, two participants were excluded due to more severe motor symptoms invalidating task performance, highlighting the challenge of independently assessing response inhibition in later disease stages. Further, while the protocol parameters (e.g., task length, number of measures) were designed to capture a wide range of possible neurophysiological changes over response withholding and inhibition, the effects of fatigue and daytime sleepiness - both common in PwPD^75,76^ - may have affected our behavioural and neurophysiological outcomes^77,78^. For instance, the EMG signal often weakened as the task progressed, likely due to muscle weakness/fatigue characteristic of PD, as it was often most pronounced in the affected side. However, it is notable that, despite reduced signal strength, we still observed increased FDI muscle bursting in PwPD, indicating that premature and partial muscle bursts are robust makers for future use in this clinical population. Building on the current study, future research could implement shorter, more tolerable protocols that directly target the neurophysiological markers identified as sensitive to inhibitory dysfunction in PwPD, thereby minimising fatigue-related confounds and accommodating more advanced motor symptoms.

Unlike much of the existing research using MEP amplitude to assess CME, we did not exclude trials based on EMG activity immediately before the TMS pulse, as these timepoints necessarily overlapped with the expected timing of muscle bursts. Therefore, automatically removing trials from the CME analysis which contained potential muscle bursts of primary interest would have weakened our ability to link CME fluctuations and muscle bursting (hypothesis 3). We instead rejected trials which showed high EMG activity (> 15 μV) 300–400 ms into the trial, prior to any expected bursting activity, and compared pre-trigger muscle activity in accordance with the CME analysis. The number of muscle bursts which coincided with the stimulation were low (HCs: 6.5 ± 5.0; PwPD: 7.7 ± 8.2 bursts) and did not significantly differ between groups (*p* = 0.602). While pre-trigger rmsEMG values rose in HCs during response inhibition – likely reflecting the stronger EMG signal in this group alongside the high incidence of trials with partial bursts – these changes did not correspond with the observed fluctuations in MEP amplitude. Future research should be cautious applying ‘blind’ pre-trigger rmsEMG exclusion criteria when integrating CME and muscle burst data, as meaningful burst-presenting trials may be inadvertently removed.

In summary, the current study identified objective neurophysiological measures sensitive to impulse control difficulties in PD. Our results corroborate previous literature demonstrating impaired response inhibition in PwPD, alongside novel results showing reduced modulation of muscle activity and CME. Our findings further extend the literature by revealing evidence of premature neuromuscular activation during response withholding, resulting in more variable behavioural responses. Collectively, the current study provides neurophysiological evidence of widespread deficits across both proactive and reactive inhibitory processes in PwPD. These changes manifested as impaired task performance and were associated with a more severe clinical profile. Further investigation into the predictive capability of these objective measures for the development of ICD symptoms will help establish their potential as a valuable tool in clinical practice.

## Methods

### Participants

Twenty HCs and 23 PwPD on ropinirole were recruited as part of a larger pre-registered study (see 10.17605/OSF.IO/KC2H3 and 10.17605/OSF.IO/W2FHP). While the pre-registered study was designed to isolate the effect of medication with the inclusion of a de novo PD group, the hypotheses and comparisons reported here focus specifically on HCs versus PwPD taking ropinirole. One PwPD was excluded due to cognitive impairment, while three PwPD and two HCs were excluded at the data analysis stage (see Results). Therefore, 18 HCs (69.1 ± 5.6 years, 9 female, 16 self-reported as right-handed) and 19 individuals (64.1 ± 7.0 years, 8 female, 16 self-reported as right-handed) with a clinical diagnosis of PD (Hoehn and Yahr stages 1–2.5) contributed data to the study. A minimum group size of 16 was determined via power analysis (α = 0.05, 80% power) based on a medium effect size (Cohen’s *d* = 0.69) reported in prior CME research^33^.

HCs were recruited via the Birmingham 1000 Elders group and PwPD via the Parkinson’s UK research network and routine NHS clinic appointments across England, Wales and Scotland. Participants were recruited if aged 40–80, with no history of neurological conditions other than PD or reported vision impairment that was not corrected (e.g., with glasses). Participants were screened for TMS eligibility based on current international safety guidelines^79^ and required a score >23 on the Montreal Cognitive Assessment (MoCA)^80^. No PwPD had received deep brain stimulation. The Movement Disorder Society-Sponsored Revision of the Unified Parkinson’s Disease Rating Scale (MDS-UPDRS; parts I-III)^81^, assessed symptom severity while PwPD were on their usual medication. The Hoehn and Yahr scale assessed functional disability^82^.

All participants gave written informed consent and received monetary compensation for their participation. The study received favourable opinion from an NHS Research Ethics Committee (South-East Scotland REC 02, IRAS ID: 328075) and was conducted in accordance with the Declaration of Helsinki.

### Behavioural task

**Fig. 6:**
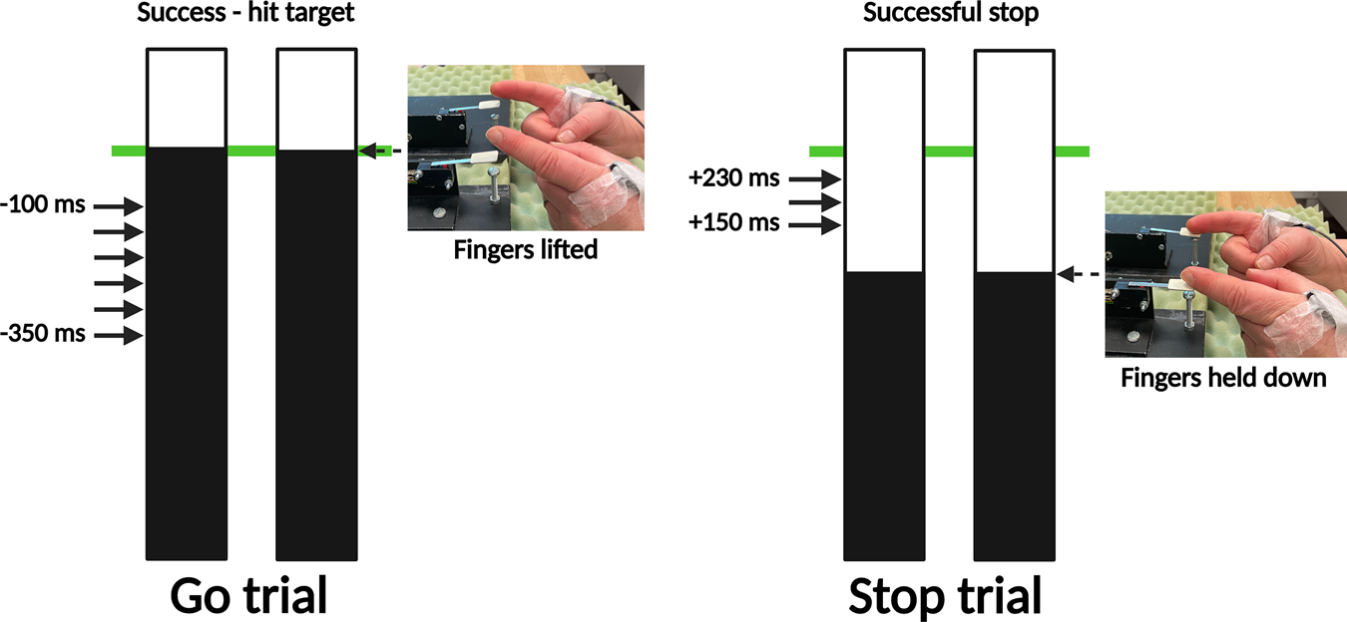
The Anticipatory Response Inhibition Task. During Go trials, participants initially held their index fingers on the respective switches and were instructed to lift their fingers once rising indicators reached the target line (at 800 ms). During Stop trials, the indicators stopped prematurely before the target and participants were instructed to inhibit the prepared finger lifts. Transcranial magnetic stimulation was delivered during response withholding (Go trials) and response inhibition (Stop trials). Arrows indicate stimulation timepoints.

The current study used the bimanual version of the ARIT (Fig. 6), controlled via custom MATLAB software (R2019b, The MathWorks) and interfaced with two custom-made microswitches. Switch ‘up/down’ state was precisely recorded (< 1 ms) through an Arduino (Uno; Arduino.cc) and synchronised to the display through an analogue-digital USB interface (NI-DAQmx9.7; National Instruments). All participants were familiar with the task, having completed it in a prior session (median time between sessions: 7 days). Participants were seated ~0.6 m in front of a computer monitor displaying two vertically oriented indicators (18 cm in length, 2 cm in width, 2 cm apart) (Fig. 6). Participants rested their forearms on a table, positioned mid-way between supination and pronation. The medial aspect of each index finger was used to lightly depress the two switches (index finger adduction), with the left and right indicators corresponding to the respective fingers. After a 2 s delay, both indicators started to move upward from the bottom at equal rates.

The task was primarily composed of Go trials (67%, 320 trials), where participants released both switches (index finger abduction) to intercept the rising indicators with a stationary target line at 800 ms. During Stop trials (33%, 160 trials), both indicators stopped before reaching the target, cueing participants to inhibit the prepared bimanual movement. The 2:1 ratio ensured Go trials were the primed response. As is common practice^22^, an adaptive staircase algorithm was incorporated into Stop trials, dynamically changing the stop-signal delay (SSD) based on trial performance to keep task difficulty consistent across participants (~ 50% success).

Visual feedback was provided after each trial to promote accurate performance. Go trials were considered successful if both indicators were within ±50 ms of the target, turning the target green and displaying *Success – hit target*. Otherwise, *Missed target* was shown with a red target. This tolerance was adapted from previous work^33^ to ensure suitability for PD, who may experience delayed processing speeds^83^. If no lift response was registered, *No response* was displayed, and the trial was excluded from analyses. On Stop trials, *Stop successful* and a green target indicated successful inhibition, while *Stop failed* and a red target indicated an erroneous lift response. Visual feedback after each experimental block comprised lift-times, Go accuracy and Stop accuracy plots.

To prevent strategic slowing, participants were to lift at the target line as accurately as possible, with ongoing visual feedback. To maintain clean EMG recordings, participants were asked to rest fingers onto the switches at trial onset and activate their muscles only during lift responses. Background EMG was visually monitored, and participants were reminded to relax their fingers if required. The Arduino device triggered both the Magstim 200^2^ and Signal software to synchronise stimulation timing.

### Recording procedure

Surface EMG (Delsys, Natick, US) was recorded over the first-dorsal interosseous (FDI) muscle of each hand as the primary agonists. The ground electrode was placed on the bony prominence of either elbow. Electrode signals were amplified, filtered (20–450 Hz), and sampled at 2 kHz (Cambridge Electronic Designs 1401, Cambridge, UK) for offline analysis with Signal (CED, v. 6.05a) and custom MATLAB software (R2019b, The MathWorks). EMG was recorded for 1 s (the length of a trial) from when the indicators started to rise.

Single-pulse TMS was applied to the M1 contralateral to the non-dominant (HC) or affected (PwPD) hand using a figure-of-eight D70^2^ coil and Magstim200^2^ unit (Magstim, Dyfed, UK). These hands were chosen to maximise the likelihood of detecting relevant effects, with the affected side in PwPD more susceptible to disease-related changes, and the non-dominant hand in HCs a more valid comparison. The coil was positioned tangentially to the head with the handle directed posteriorly at a 45° angle to the midline of the head, inducing a current directed posterior to anterior in the underlying cortical tissue^84^. The optimal coil position was identified for the contralateral FDI using a suprathreshold intensity. Coil placement was recorded and monitored using Brainsight® neuro-navigation software (Rogue Research, Cambridge, US). Each participant performed a practice block of 10-50 Go trials where TMS was applied 200 ms before the target to determine an intensity for the experimental blocks which consistently produced 200–400 µV MEPs^33^.

On Go trials, TMS was delivered 22 times at each of 50 ms intervals ranging from −350 to −100 ms relative to the target (132 total) to capture the CME profile during response withholding (Fig. 6). On Stop trials, stimulation was delivered at 150, 190 and 230 ms (40 times each) following the stop-signal.

### Dependent measures

To control for background EMG noise and/or sustained muscle activity, trials with rmsEMG exceeding 15 µV 300–400 ms after the indicators started to rise were excluded from all analyses.

To assess response withholding, lift-times on unstimulated Go trials were recorded for each index finger and the mean calculated after trimming outliers (± 3 SDs)^33^. Coefficient of variation was calculated as a measure of relative lift-time variability for each side. SSRT was calculated using the integration method^85^, as an index of inhibitory control. Trimmed lift-times averaged across side for Go trials were rank ordered and the nth lift-time selected, with n obtained by multiplying the number of lift-times by the probability of a response on a Stop trial. The time at which the staircase procedure stopped the indicators to achieve 50% success (staircased SSD) was subtracted from the nth lift-time.

EMG data processing was performed using MATLAB (MathWorks, R2022a). As per the study aims, ineffective bursts of muscle activity (i.e., those that did not trigger a lift response) were identified on a trial-by-trial basis across Go and successful Stop trials. Muscle bursts were automatically detected using a single-threshold algorithm^86^ when smoothed rectified EMG surpassed 15 SD of the baseline (0–400 ms rmsEMG) and remained elevated for ≥5 ms. This 15 SD detection threshold was empirically determined to reliably detect muscle bursts and robustly account for both low baseline magnitude in HCs as well as PD-related increases in muscle tone (e.g., tremor, rigidity). Bursts separated by <15 ms were automatically merged. Detected bursts were visually inspected on a trial-by-trial basis. While this step may have introduced subtle experimenter bias, initial burst detection was automated, and manual inspection was only conducted to remove erroneous classifications. Cases where the 15 SD threshold erroneously classified stimulus artifacts or MEPs as muscle bursts were removed, and bursts which overlapped with the stimulation (contaminated bursts) were excluded from subsequent analyses. For each burst, onset/offset, duration and peak amplitude were recorded.

On Go trials, the main burst generating the lift response was identified as the last burst with an onset before the recorded switch release. Accordingly, *premature bursts* were identified as suprathreshold EMG activity prior to the main lift response. On successful Stop trials, *partial bursts* represented muscle activity which decreased in amplitude before generating sufficient force to trigger the lift response. Bursts occurring within an individualised ‘box-of-interest’ (± 3 SD of trimmed Go lift-times) were classed as partial bursts, indicating preparation to respond. CancelTime was calculated as the difference in milliseconds between the SSD and the peak amplitude of the partial EMG burst (e.g., the point at which muscle activation begins to decrease)^52^. To index burst incidence, burst trial incidence (% of traces with ≥1 burst(s)), burst frequency (mean bursts per bursting trace) and cumulative burst rate (incidence × frequency) were calculated for premature and partial bursts. All bursting measures were also recorded separately for each hand for comparison between more and less affected sides.

CME was measured via peak-to-peak MEP amplitude in the FDI of the non-dominant (HC) or affected (PD) hand. To ensure consistent TMS delivery, stimulated trials where the coil deviated more than 3 mm/5° from the target position were excluded. At each stimulation time, MEPs were trimmed by excluding the upper and lower 10% (if ≥10 MEPs; met in all but 7/162 HC and 18/171 PwPD data points) for a more accurate measure of centrality^87^, as previously described^33^. Pre-trigger muscle activity was calculated via rmsEMG 5–55 ms prior to the TMS pulse.

The Barratt Impulsiveness Scale (BIS-11)^88^ assessed trait impulsivity, reflecting an individual’s tendency to act on impulses rather than engage in considered decision-making, across three broad domains: attentional impulsiveness (difficulty focusing and shifting attention), motor impulsiveness (acting without thinking), and non-planning impulsiveness (lack of future-oriented thinking). Participants rate each item on a 4-point Likert scale (1 = rarely/never, 4 = almost always). Of 30 items, two job-related questions were excluded as participants were often retired. Therefore, total scores ranged from 28 to 112, where higher scores indicate greater impulsivity.

The Questionnaire for Impulsive-Compulsive Disorders in Parkinson’s Disease Rating Scale (QUIP-RS) is a validated 28-item self-report measure for ICD behaviours in PD^89^, covering four primary ICDs–pathological gambling, compulsive buying, hypersexuality, and binge eating–as well as three related behaviours: punding, hobbyism, and dopamine dysregulation syndrome. Each item is rated on a 5-point Likert scale (0 = never, 4 = very often), with higher scores indicative of greater ICD symptomology. HCs did not respond to four questions specific to PD medication (maximum score of 96 versus 112). Total scores were converted to percentages to enable between-group comparison.

### Statistical analysis

No interim group level analyses were performed during data collection. The following analyses were conducted on the final dataset. Participant demographic, cognitive and self-report impulsivity measures were compared between groups (Table 1). Kolmogorov–Smirnov tests identified any violations of normality. Non-normal data were log-transformed prior to being entered into an ANOVA. Post-hoc comparisons were performed where necessary, with statistical significance set at α = 0.05 and adjusted for multiple comparisons using the Sidak correction. Greenhouse-Geisser p-values are reported for violations of sphericity. Results are presented as group means ± SD.

To determine whether PwPD demonstrated impaired response withholding (hypothesis 1a), lift-times (mean, coefficient of variation) were examined in a 2 × 2 mixed-effects ANOVA with factors Hand (more affected/non-dominant, less affected/dominant) and Group (PwPD, HC). To examine response inhibition performance (hypothesis 1b), between-group comparisons of SSD, SSRT and CancelTime were conducted using one-tailed independent-samples t-tests. CancelTime was included post-registration as a novel index of response inhibition recently validated with the ARIT^26^. To assess the level of agreement between these indices (standard SSRT vs. novel CancelTime), a Pearson correlation analysis was conducted.

To examine muscle bursting patterns during response withholding (hypothesis 2a), the following premature burst characteristics were examined between groups using a multivariate ANOVA: burst trial incidence, burst frequency, cumulative burst rate, amplitude and duration. The same analysis was performed on partial bursts to explore muscle bursting dynamics during response inhibition (hypothesis 2b). Bursting characteristics that differed between groups were further compared between the more and less affected sides of PwPD using two-tailed paired-samples *t*-tests.

To examine fluctuations in CME while a response was withheld (hypothesis 3a), a 2-Group × 6-Stimulation Time (−350, −300, −250, −200, −150, −100 ms relative to target) mixed ANOVA was conducted on mean MEP amplitude in Go trials. To investigate CME fluctuations while a response was inhibited (hypothesis 3b), a 2-Group × 3-Stimulation Time (150, 190, 230 ms relative to stop-signal) mixed ANOVA analysed MEP amplitude on successful Stop trials. To ensure pre-trigger muscle activity did not influence CME changes, the analyses were repeated with pre-trigger rmsEMG values. Additional LMMs were applied to confirm the reliability of the mixed ANOVAs performed on lift-times and CME while accounting for individual variability (see Supplementary Material).

To explore potential associations between neurophysiological measures, task performance and self-reported impulsivity (hypothesis 4), stepwise linear regression models were applied. Stepwise regression was employed to reduce the risk of overfitting relative to the number of predictors. Predictor variables were selected based on significant group comparisons. Regression models were performed on log-transformed data to meet normality and homoscedasticity assumptions.

## Supplementary information

Supplementary Information

## Data Availability

The anonymised datasets analysed in the current study are available on the Open Science Framework: 10.17605/OSF.IO/3XWDA.

## Code availability

The custom MATLAB scripts for the current study are available on the Open Science Framework: 10.17605/OSF.IO/3XWDA.
