# Supplementary material for: Muscle bursting and corticomotor excitability mark impaired impulse control in Parkinson’s disease
Source: NPJ Parkinsons Dis. 2025 Dec 23;12:11. doi: 10.1038/s41531-025-01207-5 (PMC12796308; doi:10.1038/s41531-025-01207-5)
Supplement: Supplementary file 1 — Supplementary Information [file 41531_2025_1207_MOESM1_ESM.pdf]

Supplementary Material

Linear mixed models

Linear mixed-effects models were performed on behavioural (Go lift-times and coefficient of variation) and CME (log-transformed Go and Stop trial MEP amplitudes) data using the lme4 package in R (Version 4.4.1). The behavioural models included Group (HC, PwPD) and Hand (more affected/non-dominant, less affected/dominant) and their interaction as fixed effects, age as a covariate, and participant as a random intercept. The CME models substituted Hand with Stimulation Time (-350, -300, -250, -200, -150, -100 ms relative to the target on Go trials; 150, 190, 230 ms relative to the stop-signal on successful Stop trials).

For lift-times, there was a significant Group × Hand interaction (E = 0.013, $p < .001$), with the more affected hand (837.2 ± 24.7 ms) lifting later than the less affected hand (824.5 ± 22.5 ms, $p < 0.001$) in PwPD. There was no main effect of Group (E = 0.002, $p = .721$) or Hand (E = 0.001, $p = .249$). For coefficient of variation, there was a main effect of Group (E = 0.944, $p = .006$) with a higher coefficient of variation in PwPD, but no effect of Hand (E = 0.075, $p = .460$) or interaction (E = 0.158, $p = .265$).

For corticomotor excitability on Go trials, there was a main effect of Stimulation Time, with CME increasing relative to baseline (-350 ms) in both groups from -250 ms onwards (all E > 0.398, all $p < .001$). There was no main effect of Group (E = 0.126, $p = .634$) on CME. However, a Group × Stimulation Time interaction revealed that, relative to their baseline MEP amplitudes at -350 ms, PwPD showed a greater increase in CME at -200 ms (E = 0.573, $p < .001$) and -150 ms (E = 0.615, $p < .001$) relative to HCs.

For Stop trials, there was a main effect of Stimulation Time, with reduced CME at 190 ms (E = 0.829, $p < .001$) and 230 ms (E = 0.890, $p < .001$) relative to 150 ms after the stop-signal.

While there was no main effect of Group (E = 0.019, p = .417), a Group × Stimulation Time interaction revealed a smaller drop in CME relative to 150 ms at both 190 ms (E = 0.273, p = .014) and 230 ms (E = 0.257, p = .017) for PwPD.
